# Choice of immobilization of stereotactic body radiotherapy in lung tumor patient by BMI

**DOI:** 10.1186/s12885-019-5767-1

**Published:** 2019-06-14

**Authors:** Guofu Chen, Baiqiang Dong, Guoping Shan, Xiuqin Zhang, Huarong Tang, Yuchen Li, Zhenhua Wang, Wei Xu, Gang Xu, Guiming Yan, Feiyan Zhang, Xiao Hu, Jun Yang, Yujin Xu, Ming Chen, Jin Wang

**Affiliations:** 1 Department of Radiation Oncology, Zhejiang Cancer Hospital, 1st Banshan East Road, Hangzhou, 310022 China; 2 Zhejiang Provincial Key Laboratory of Radiation Oncology, Hangzhou, 310022 China; 3 People’s hospital of Yuxi city in Yunnan province, Yuxi, 653100 China; 4 Yitu Healthcare, Shanghai, China

**Keywords:** Lung cancer, SBRT, Interfraction motion, Immobilization, BMI

## Abstract

**Background:**

An accurate, reproducible, and comfortable immobilization device is essential for stereotactic radiotherapy (SBRT) in patients with lung cancer. This study compared thermoplastic masks (TMP) and vacuum cushion (VCS) system to assess the differences in interfraction and intrafraction setup accuracy and the impact of body mass index (BMI) with respect to the immobilization choice.

**Methods:**

This retrospective study was conducted on patients treated with lung SBRT between 2012 and 2015 at the Zhejiang cancer hospital. The treatment setup accuracy was analyzed in 121 patients. A total of 687 cone beam computed tomography (CBCT) scans before treatment and 126 scans after treatment were recorded to determine the uncertainties, and plan target volume margins. Data were further stratified and analyzed by immobilization methods and patients’ BMI. The t-test (Welch) was used to assess the differences between the two immobilization systems when stratified by the patients’ BMI.

**Results:**

For patients with BMI ≥ 24, the mean displacements for the TMP and VCS systems were 1.4 ± 1.2 vs. 2.4 ± 2.0 mm at medial-lateral (ML) direction (*p* < 0.001); 2.0 ± 1.9 vs. 2.0 ± 1.9 mm at cranial-caudal (CC) direction (*p* = 0.917); and 2.4 ± 1.4 vs. 2.6 ± 2.1 mm at anterior-posterior (AP) direction, (*p* = 0.546). The rate of acceptable errors increased dramatically when immobilized by TMP. In the case of patients with BMI < 24, the mean displacements for the TMP and VCS systems were 1.8 ± 1.4 vs. 2.1 ± 1.8 mm at ML direction (*p* = 0.098); 2.9 ± 2.3 vs. 2.2 ± 2.2 mm at CC direction (*p* = 0.001); and 1.8 ± 1.8 vs. 2.3 ± 2.0 mm at CC direction, (*p* = 0.006). The proportion of acceptable errors increased after immobilization by VCS. No difference was detected in the intrafraction setup error by different immobilization methods.

**Conclusions:**

The immobilization choice of SBRT for lung tumors depends on the BMI of the patients. For patients with BMI ≥ 24, TMP offers a better reproducibility with significantly less interfractional setup displacement than VCS, resulting in fewer CBCT scans. However, VCS may be preferred over TMP for the patients with BMI < 24. Therefore, an optimal immobilization system needs to be considered in different BMI groups for lung SBRT.

## Background

In recent years, multiple studies demonstrated that the overall survival of patients undergoing stereotactic body radiation therapy (SBRT) is similar to that of surgical resection in operable stage I non-small cell lung cancer (NSCLC) [1–3]. In addition, for inoperable early-stage lung cancer, SBRT is a critical alternative therapy [4]. Also, it is one of the major local therapies for oligometastatic lung tumors [5]. SBRT is characterized with few treatment fractions and high-dose in each treatment. The accurate image guidance restricted the high-dose radiation to the target areas, while doses outside the target areas declined rapidly to avoid damage to the critical adjacent organs [6–8].

To reduce the tissue toxicity maximally and ensure the accuracy of implementation, currently four-dimensional computed tomography (4D-CT) is used to eliminate the effects of respiratory motion. Subsequently, the PTV margins are minimized, and setup errors between fractionated radiations are corrected using cone-beam CT (CBCT) in the premise of accurate radiation on the target areas. The setup errors in the medial-lateral (ML) direction, the cranial-caudal (CC) direction, and the anterior-posterior (AP) direction are limited within 2–5 mm. If the positional discrepancies are substantial, CBCT verification should be repeated after the couch adjustment [9]. Therefore, the repetitive CBCT scans extend the overall treatment duration, along with additional cost and workload of the technicians [10]. The longer the treatment duration, the more likely the displacements in patients to occur during SBRT [11]. However, the interfractional setup errors and intrafractional motions should be avoided. In addition to the factors such as co-operation of patients and the experience of technicians, the selection of immobilization method is also crucial [12].

Thermoplastic masks (TMP) and vacuum cushions (VCS) are commonly used immobilization devices in SBRT; however, there are only a few studies comparing the two devices. Navarro-Martin et al. demonstrated that the setup errors of the two devices TMP were smaller. Markedly, the TMPs were favored by the technologists [10]. Nevertheless, the body shape and physical condition of the patients, as well as compliance with the advice of the doctors differed in a practical setting. Thus, one immobilization device might not be suitable for all patients, and different devices might be used based on the characteristics of the patients.

Therefore, the present study assessed the interfraction and intrafraction setup errors by different immobilizations in patients with lung tumors who underwent SBRT. Moreover, the effects of patient-related factors and immobilization devices on the interfraction and intrafraction setup errors were analyzed. Herein, we hypothesized that the immobilization devices were selected based on the characteristics of patients in order to ensure the accurate implementation of SBRT.

## Methods

### Patient data

The present study was approved by the Ethics Committee and Institutional Review Board of Zhejiang Cancer Hospital (belongs to Zhejiang Cancer Hospital; the committee’s reference Number: IRB-2018-153(Ke)). The medical records of NSCLC patients, who underwent SBRT in Zhejiang Cancer Hospital from January 2012 to September 2015 were reviewed. The inclusion criteria stated that the patients with early-stage (T1-T2N0M0) NSCLC lung cancer or oligometastatic lung cancer (IVa stage) were eligible for participation in this study. NSCLC (including carcinoma, adenocarcinoma, large cell carcinoma, and mixed cell carcinoma) in all patients was confirmed by histology. However, patients with several tumors in the lung were excluded from the analysis.

### Radiotherapy

Patients were immobilized by TMPs (Klarity Medical Products, Newark, NJ, USA) or VCSs (Klarity Medical Products, Newark, NJ, USA) [13]. The patients were positioned in the immobilization device with the arms placed above the head. In the simulation, free breathing and 4D-CT (Philips Brilliance CT Big Bore, USA) were undertaken. The scan encompassed the upper margin of the second cervical spine up to the lower margin of the second lumbar spine with 3–5 mm layer thickness. The inhalation and exhalation correlated datasets were identified and transferred to the treatment planning system (RayStation Launcher 4.5.1, RaySearch Laboratories AB, Sweden or Philips Pinnacle 9.2 treatment planning system, Amsterdam, The Netherlands) for delineation of the gross tumor volume (GTV). The time phase interval of respiration in 4D-CT scanning was 10% with 10 images of the respiratory time phase on each layer. Furthermore, lung window and mediastinal window were compared. The GTV was contoured phase-by-phase referring to the result of chest CT or PET/CT. No margin was applied to the GTV to generate the clinical target volume (CTV). The GTV inhale and GTV exhale were fused to generate the internal tumor volume (ITV). The planning target volume (PTV) expanded 5–8 mm in every direction based on the ITV range. Moreover, organs at risk (OAR) including spinal cord, bilateral lungs, trachea, chest wall, brachial plexus, heart, and esophagus were contoured. The conformality and dose limits of normal tissues were set according to RTOG0236 [14]. The treatment requirements for large lesions (maximum cross-sectional diameter > 4 cm) or adjacent vital organs were satisfied by reducing the dose in each fraction and increasing the number of fractions. In the treatment, 80% iso-dose line was used as the prescribed dose to cover 95% PTV, and 100% iso-dose line was used to cover 100% ITV. The fraction dose was 5–15Gy. Patients received 4–10 SBRT fractions (mean = 5) by 6MV X-ray in 6–14 fields using the coplanar technique IMRT or volumetric modulated arc therapy (VMAT) (ElektaSynergy™, Stockholm, Sweden) or Varian Trilogy-SN5387 linear accelerator (Varian Medical Systems Inc., Palo Alto, CA, USA).

### CBCT and data collection

For initial treatment, the patients were set up to isocenter with in-room lasers and skin tattoos. Then, CBCT scanning was conducted in a 360° standard rotation mode. First, a rigid registration of the bony anatomy was performed to assess rotation, initiating the patient re-setup by therapists if a ± 3° rotational tolerance was exceeded. The manual registration of the ITV contour was performed with respect to patient’s tumor and soft-tissue target after the rotational parameters were measured within the tolerance limits. The data on the shifts were obtained from On Bio-rad Imager 1.6 system (Varian Medical Systems Inc., Baden, Switzerland), and cross-sectional, sagittal, and coronal CT images were obtained by analysis and reconstruction (automatic registration function equipped with OBI 1.6 software). The grayscale registration was conducted on areas including the tumor, the surrounding tissue, and vital organs at risk. The shifts from the first couch were adjusted accordingly. The tolerance of CBCT for treatment at our institute was 5, 5, and 5 mm in left-right, AP, and CC directions, respectively. Those exceeding these limits required verification of CBCT when the shift was adjusted. The couch position from the first fraction treatment was used as the couch position for the other fractions. Subsequently, the treatment was performed, post-treatment CBCT scanning was conducted after the treatment, and intrafraction setup errors data were obtained.

### Statistical analysis

The setup errors included translational errors in the ML direction, CC direction, and AP direction. The mean and standard deviation of the translational errors were calculated. The multiple groups were compared by analysis of variance (ANOVA), while the pairwise comparison of the mean was conducted by LSD test. The difference in the ratio was compared by chi-square test. The expanded value from ITV to PTV was calculated by the expansion equation of the CTV to PTV as proposed by van Herk: PTV = 2.5 × mean + 0.7 × standard deviation (13), which was considered to be statistically significant if bilateral *P* < 0.05. All data were analyzed using SPSS (Statistics version 21, SPSS Inc. IBM, Chicago, IL).

## Results

### Correlation between interfraction setup errors and clinical factors

The 687 CBCT scans were collected from a cohort of 121 patients. In total, 60 patients received IMRT, while 61 patients received VMAT. Subsequently, 26 patients received 12.5Gy × 4 fractions, 91 received 10Gy × 5 fractions, 1 received 7.5Gy × 8 fractions, 2 received 7Gy × 10 fractions, and 1 received 6Gy × 10 fractions. The interfraction setup errors of patients are shown in Table 1.

**Table 1:** Summary of positional error stratified by patient related factors

Patient related factor	N(Pts)	Positional error (mean ± deviation, mm)		
		ML	CC	AP
Age				
≥ 70	57	1.9 ± 1.8	2.4 ± 2.3	2.3 ± 2.0
< 70	64	2.1 ± 1.7	2.4 ± 2.2	2.2 ± 2.0
Primary sites				
Left upper lobe	33	2.0 ± 1.8	2.7 ± 2.5	2.1 ± 2.0
Left lower lobe	17	1.8 ± 1.7	2.9 ± 2.5	2.1 ± 1.8
Right upper lobe	29	2.0 ± 1.6	1.9 ± 1.6	2.4 ± 2.2
Right middle lobe	18	1.4 ± 1.4	2.4 ± 2.3	2.1 ± 1.7
Right lower lobe	24	2.4 ± 1.8	2.2 ± 1.9	2.0 ± 1.8
Education				
Illiterate	39	2.0 ± 1.9	2.1 ± 2.0	2.2 ± 2.0
Primary school	37	1.9 ± 1.6	2.1 ± 2.0	2.4 ± 2.1
Middle school	28	1.9 ± 1.7	2.6 ± 2.3	2.0 ± 1.5
High school	8	2.7 ± 2.1	3.0 ± 2.3	2.8 ± 2.5
University qualifications	9	2.3 ± 1.6	3.2 ± 2.8	2.7 ± 2.2
Urban/Rural				
Urban	54	2.2 ± 1.8	2.4 ± 2.3	2.4 ± 2.0
Rural	67	1.9 ± 1.7	2.3 ± 2.1	2.2 ± 1.9
KPS				
> 80	84	2.0 ± 1.5	2.2 ± 2.3	1.1 ± 1.0
≤ 80	17	1.5 ± 1.9	2.1 ± 2.0	2.2 ± 1.9
Gender				
Male	88	2.0 ± 1.8	2.1 ± 2.0	2.3 ± 2.0
Female	33	1.9 ± 1.7	3.1 ± 2.6	2.3 ± 1.8
Immobilization				
Thermoplastic masks	43	1.6 ± 1.3	2.8 ± 2.3	2.0 ± 1.7
Vacuum cushion	78	2.2 ± 2.0	2.2 ± 2.1	2.4 ± 2.1
BMI				
BMI < 18.5	16	1.9 ± 1.7	2.3 ± 2.1	2.0 ± 1.7
18.5 ≤ BMI < 24	65	2.0 ± 1.7	2.5 ± 2.3	2.1 ± 2.0
BMI ≥ 24	40	2.1 ± 1.8	2.0 ± 1.9	2.6 ± 1.9

Abbreviations: Pts = patients; ML = medial–lateral; CC = cranial–caudal; AP = anterior–posterior

As shown in Table 1, no correlation was established in the maximum interfraction setup errors in any direction irrespective of the factors such as the age, educational status, lesion location (left upper lobe, left lower lobe, right upper lobe, right middle lobe, right lower lobe), patients from rural or urban areas, body mass index (BMI), Karnofsky performance status (KPS), and methods of immobilization.

A rigid registration of the bones was performed to assess the patient’s rotation error. A total of 228 rotational errors were recorded. The possibility of exceeding the rotational tolerance (> 3°) was 0.6% for those immobilized by TMP, and 2.0% for by VCS. However, no difference was detected while using different immobilization devices (*P* = 0.398, OR 0.28, 95% CI: 0.02–4.62).

**Fig. 1:**
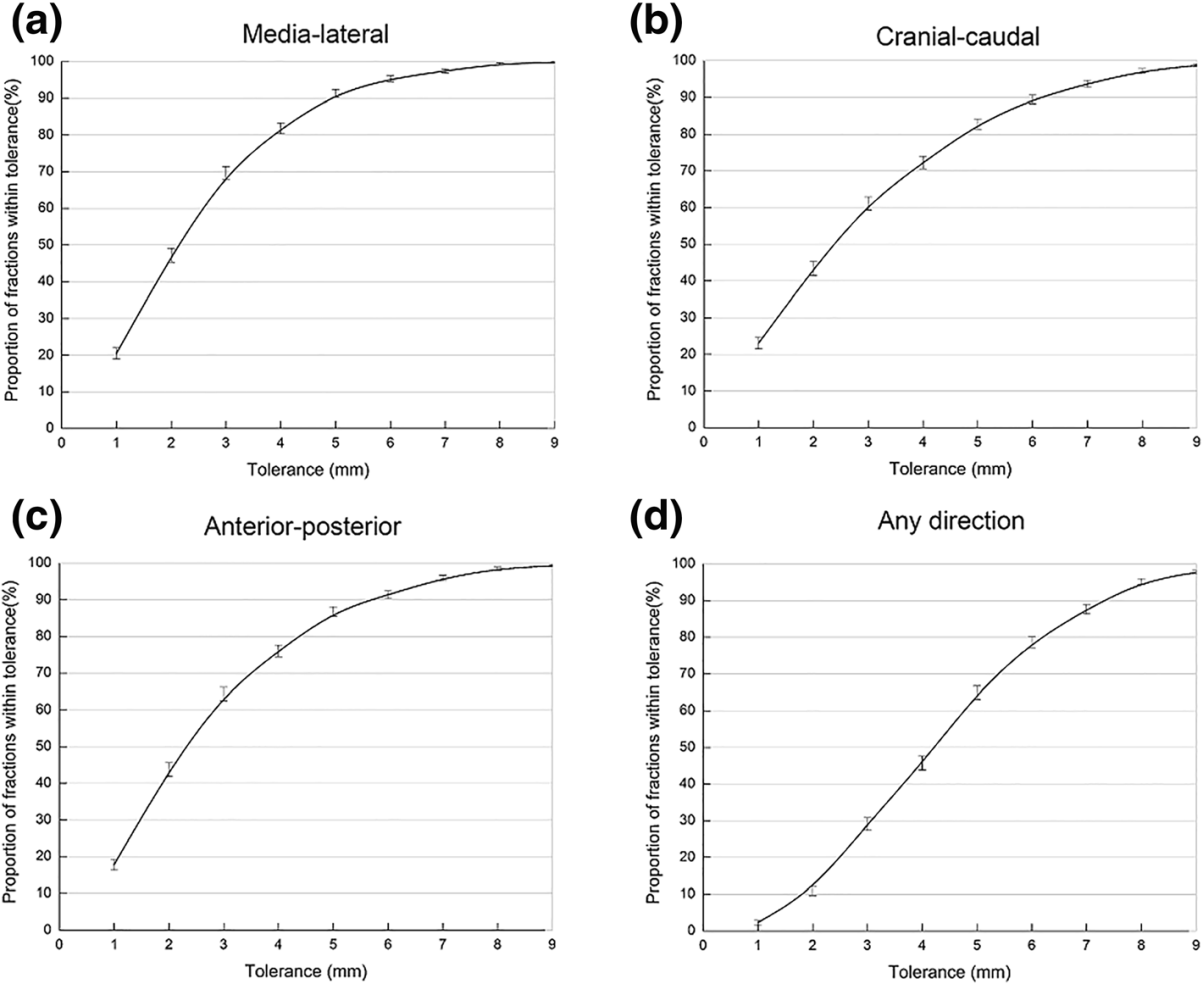
Proportion of treatment fractions within tolerance measured based on the cone-beam computed tomography (CBCT) scans in **a** medial-lateral direction, **b** cranial-caudal direction, **c** anterior-posterior direction, **d** any direction (*N* = 121 patients)

Thus, the setup errors in the ML, CC, and AP directions were less than a specific value, otherwise, CBCT necessitates verification. For example, if the CBCT required verification when the setup error was > 5 mm, then that in the ML, CC, and AP directions satisfying the initial CBCT< 6 mm were 95.3, 89.5, and 91.4%, respectively. The setup error < 6 mm in any direction was potentially 78.6%, while 21.4% was the probability of the necessity of CBCT verification (Fig. 1).

### Interfraction setup errors stratified by immobilization and BMI

**Fig. 2:**
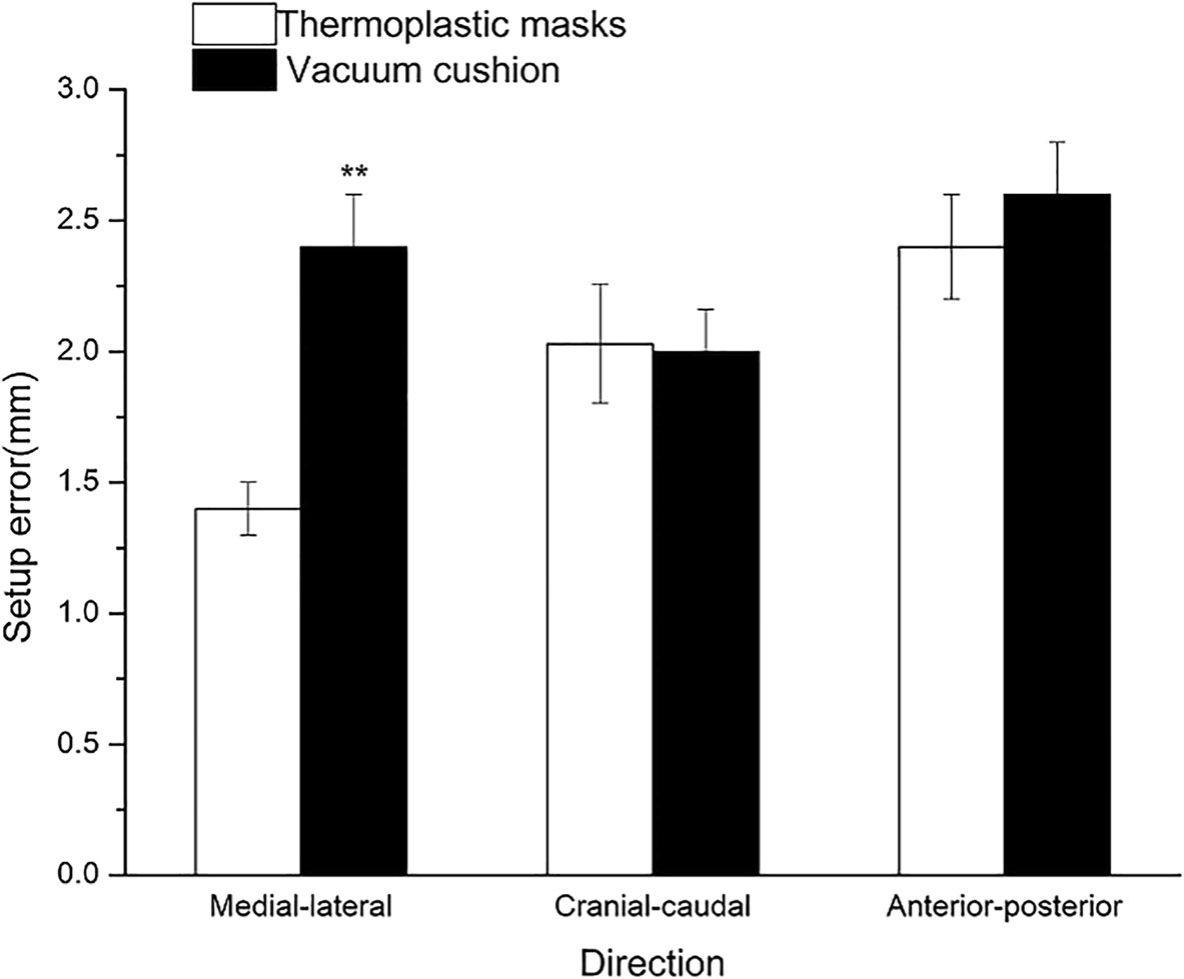
Set-up error stratified by immobilization type in patients with BMI ≥ 24

The patients were divided into two groups: BMI < 24 and BMI ≥ 24. The effects of different ways of immobilization on the interfraction setup errors after grouping were compared and the probability of CBCT verification was calculated. In the group with BMI ≥ 24, the setup error in the ML direction immobilized by TMP was 1.4 ± 1.2 mm, which was lower than that of VCS (2.4 ± 2.0 mm) (*P* < 0.001). The setup errors in the CC and AP directions immobilized by TMP were 2.0 ± 1.9 mm and 2.4 ± 1.4 mm, respectively, which did not differ significantly as compared to those by VCS (2.0 ± 1.9 mm in the CC direction, *P* = 0.917; 2.6 ± 2.1 mm in the AP direction, *P* = 0.546; Fig. 2).

**Fig. 3:**
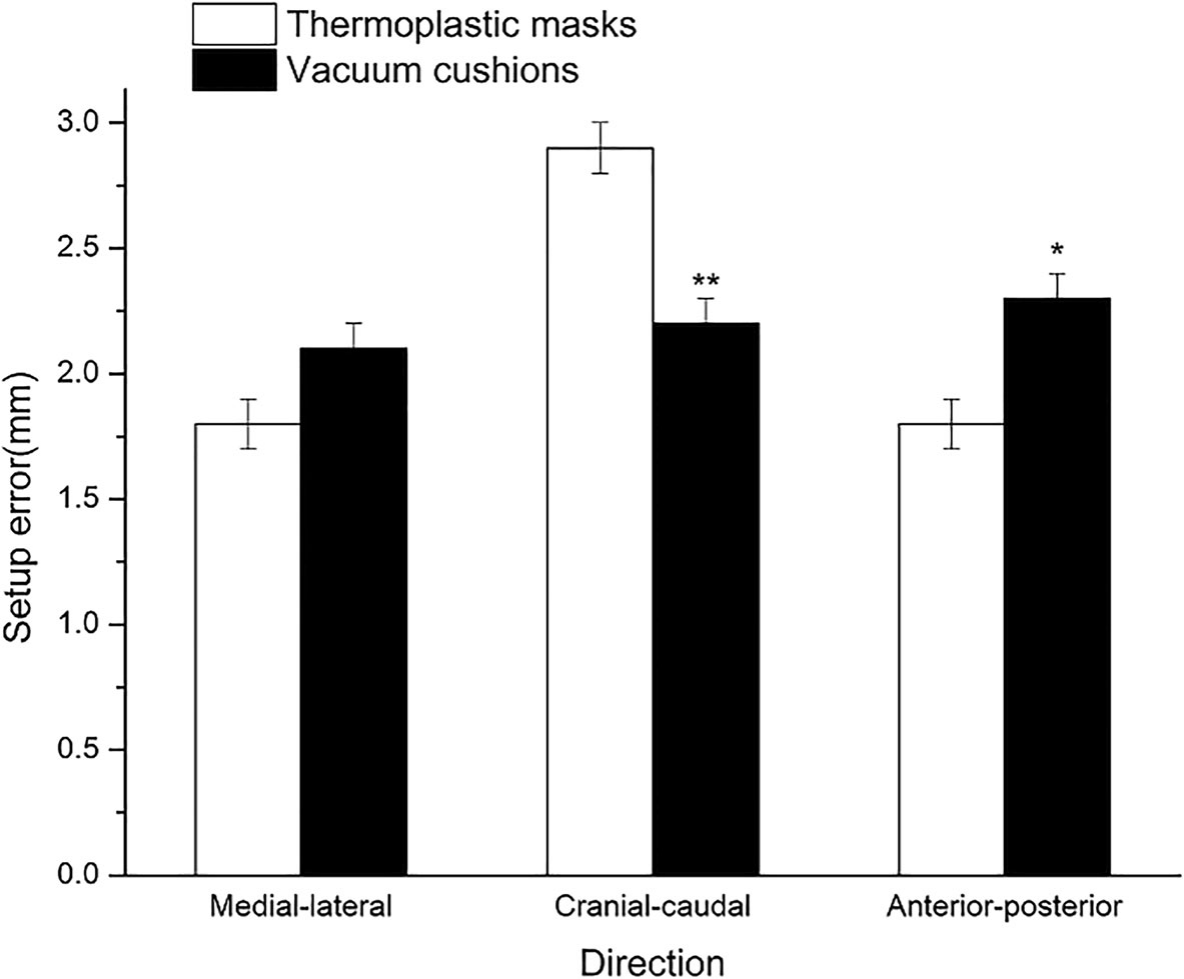
Set-up error stratified by immobilization type in patients with BMI < 24

In the group with BMI < 24, the interfraction setup error in the CC direction immobilized by VCS was 2.2 ± 2.2 mm, which was significantly lower as compared to the TMP (2.9 ± 2.3 mm; *P* = 0.001). However, the interfraction setup error in the AP direction immobilized by TPM was 1.8 ± 1.8 mm, which was significantly lower as compared to that by VCS (2.3 ± 2.0 mm; *P* = 0.006). Furthermore, no significant difference was observed in the ML direction between TMP and VCS (1.8 ± 1.4 mm in the CC direction vs. 2.1 ± 1.8 mm in the AP direction; *P* = 0.098; Fig. 3).

**Fig. 4:**
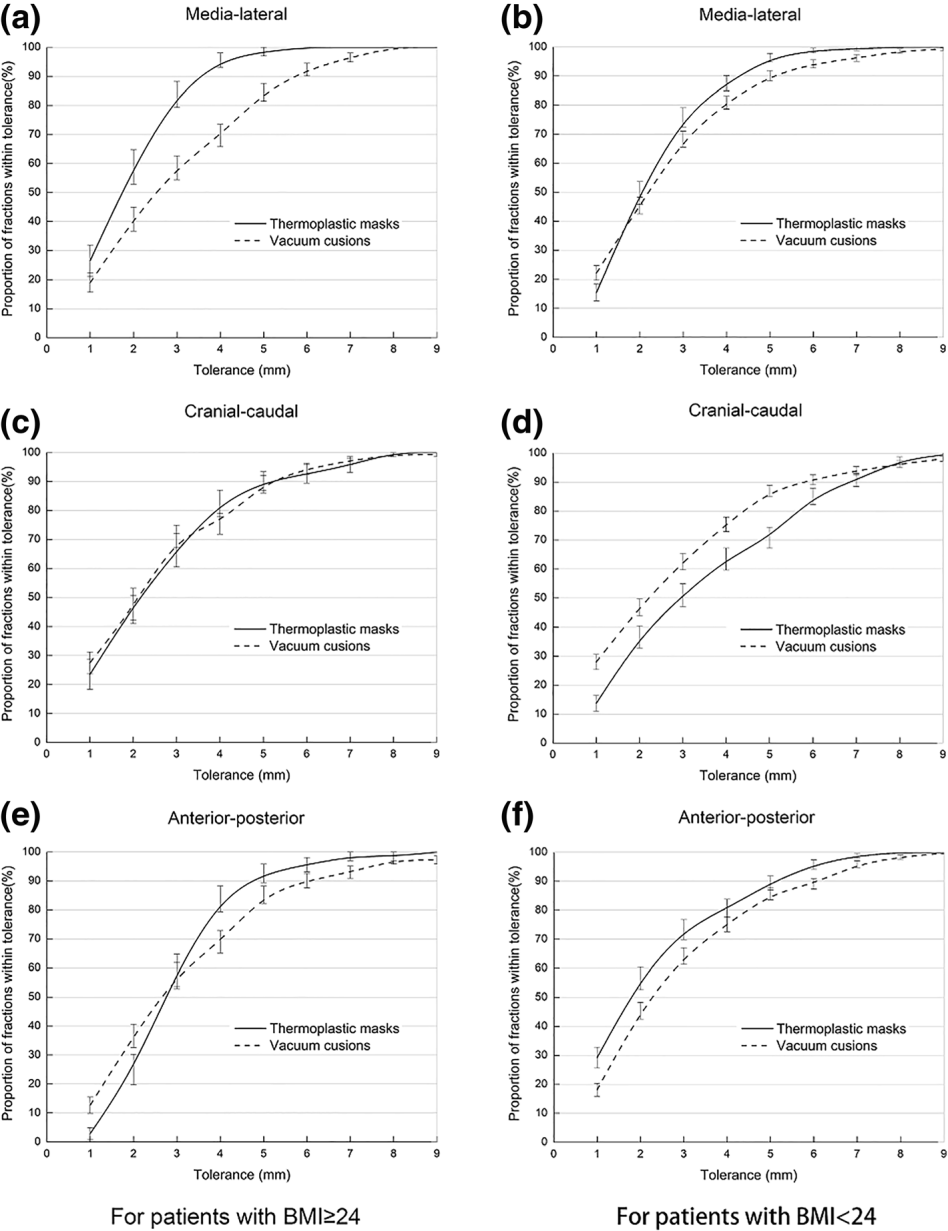
Proportion of treatment fractions within tolerance stratified by immobilization type and BMI. **a** Medial-lateral direction in patients with BMI ≥ 24; **b** Medial-lateral direction in patients with BMI < 24; **c** Cranial-caudal direction in patients with BMI ≥ 24; **d** Cranial-caudal direction in patients with BMI < 24; **e** Anterior-posterior direction in patients with BMI ≥ 24; **f** Anterior-posterior direction in patients with BMI < 24 (*N* = 121 patients)

Furthermore, the acceptable setup errors in each direction did not exceed a specific value, or else, CBCT would require verification. The correlation between acceptable errors and rate in the ML, CC, and AP direction immobilized by VCS or TMP is shown (Fig. 4).

### Intrafraction setup error

A total of 126 CBCT scans from 29 patients before and after radiotherapy were collected. The relative displacements before and after the treatment were 0.9 ± 1.0 mm in the ML direction, 1.1 ± 1.5 mm in the CC direction, and 1.2 ± 1.2 mm in the AP direction. The required expansions of PTV are listed (Table 2) according to different recipes. A total of 22 patients in the cohort used TMP, and the displacements during the treatment were 0.9 ± 1.0 mm in the ML direction, 1.1 ± 1.3 mm in the CC direction, and 1.2 ± 1.2 mm in the AP direction. On the other hand, 7 patients used VCS, and displacements were 1.3 ± 1.3 mm in the ML direction, 1.6 ± 2.2 mm in the CC direction, and 1.6 ± 1.3 mm in the AP direction. However, no significant difference was noted in all relative displacements of patients when different ways of immobilization were employed during the treatment (all *P* > 0.05).

**Table 2:** PTV margin changes of lung tumor patients

Author	Recipe	PTV margin(mm)		
		ML	CC	AP
Stroom et al.	2Σ + 0.7σ	2.5	3.3	3.2
van Herk et al.	2.5Σ + 0.7σ	3.0	3.8	3.8
Parker et al.	Σ+ $\sqrt{\upsigma^2+{\Sigma}^2}$	2.2	3.0	2.9
Snoke JJ	2.5Σ + β(σ2 + σP 2)^1/2^ − βσP	2.7	3.6	3.6

Abbreviations: *ML* medial–lateral, *CC* cranial–caudal, *AP* anterior–posterior

Symbols: Σ, standard deviation of systematic uncertainties; σ, standard deviation of statistical (random) uncertainties. σP =0.64, β =0.84

## Discussion

Since the SBRT fraction dose is relatively high and the dose of the surrounding normal tissues declines rapidly, the accuracy and high-dose radiation to tumor tissues, as well as the maximum protection of the surrounding normal tissues can be ensured by the SBRT setup. Therefore, an adequate immobilization device is critical, which might reduce the interfractional and intrafractional setup errors.

Nevertheless, the localization of CBCT (localization CBCT) should be performed after the patients are positioned in the treatment bed. If significant errors were found during localization CBCT, then further verification of CBCT should be conducted. During the treatment, an additional CBCT (intrafraction CBCT) may be needed to ensure the proper range of PTV for the target areas. Subsequently, the treatment is suspended if errors are detected; nonetheless, it is continued after reset. Then, a post-treatment CBCT was performed in order to assess the large displacement during the treatment [11].

However, the longer the patients lie in the treatment bed in a specific posture, the more likely are the displacements to occur. Moreover, SBRT is usually required by elderly patients with poor pulmonary functions [15, 16]; the shortening of the treatment duration is very important. In addition, abundant CBCTs increases the expense of the patients and workload of the technicians.

The widespread application of SBRT based on VMAT technology results in a 37–63% shortening of the treatment time [17, 18]. Thus, intrafraction CBCT in patients with a short treatment time might not be essential [19].

In order to reduce the frequency of verification CBCT, a majority of the centers stated that the verification CBCT should be conducted if errors are less than a specific value [9]. For example, our center postulates that the verification CBCT should be performed to revise the position, only if the bed moves greater than 5 mm [13].

The immobilization methods might affect the setup errors [19, 20]. Nevertheless, relatively few studies addressed the immobilization techniques. Several studies compared the effects of abdominal compression and VCS on SBRT setup error and did not find any difference [9, 10, 12]. Moreover, Li et al. [9] did not detect any differences while comparing the effects of abdominal compression, chest board, and VCS on setup errors. Furthermore, Navarro-Martin et al. compared the effects of TMP and VCS on SBRT and demonstrated that the setup errors of TMP were smaller and more favored by patients than VCS [10]. However, these studies did not compare the effects of different immobilization devices by combining the characteristics of the patients (physique). Patients with slim bodies (such as most Asian patients) have different feelings of comfort to TMP as compared to those with plump bodies. Although it has not yet been reported, the effects of different immobilization devices on the setup errors of patients with different physiques might be varied. To investigate the above questions of body sizes, our study designed two patient groups based on BMI < or > 24. Subsequently, the errors in various directions under different immobilization devices were analyzed between patients with slim and plump bodies. The current study found that errors (2.2 vs. 2.9, *P* = 0.001) in the CC direction were small in the case of patients with slim bodies (BMI < 24) immobilized by VCS, which might be attributed to the correlation between the hand gesture and errors in that direction. Therefore, the usage of hands was rather comfortable with superior repeatability upon the usage of VCSs. The errors in the AP direction were slightly larger (2.3 mm in VCSs vs. 1.8 mm in TMPs); however, SBRT was frequently used in elderly patients, and elderly Chinese patients with slim bodies were often accompanied with humps. Thus, such patients would be rather comfortable if VCSs were used, and hence, it is recommended for patients with slim bodies.

The current study demonstrated that immobilization effects were satisfactory if TMPs were used for patients with plump bodies (BMI ≥ 24). Compared to VCSs, the setup errors were reduced in the ML direction (1.4 vs. 2.4 mm; *P* < 0.001), while those in the CC direction were similar to the errors in the AP direction. Therefore, the possibility of a repeat verification CBCT was shortened in these patients, thereby preferring the immobilization by TMPs for patients with plump bodies.

Furthermore, the displacement during the treatment was not significantly different irrespective of VCS or TMP. Thus, it can be concluded that the displacements of patients during the treatment were not affected by the immobilization devices [21]. In summary, we proposed that different immobilization devices should be used based on the BMI of patients by analyzing the setup errors of patients with different physiques using various immobilization devices. The current study recommended the use of VCS for patients with BMI < 24 and TMP for patients with BMI ≥ 24. However, no significant difference was observed in the errors of treatment between the two immobilization masks. Nevertheless, this retrospective study also presented some limitations. Firstly, the time of each treatment was not recorded and it might also affect the displacements of patients before and after the treatment. Secondly, approximately, 67% of the data on rotational errors were missing during analysis. Next, the patient’s comfort and tolerance have not been yet considered in the present study. Although the conclusions of this study were not affected, the effects of additional factors (including treatment time and the feeling of pain) on the setup errors and immobilization devices should be investigated in order to promote a sophisticated process of SBRT and improve the service provided to the patients.

## Conclusion

The immobilization choice of SBRT for lung tumors depends on the BMI of the patients. For patients with BMI ≥ 24, TMP offers a better reproducibility; however, VCS may be preferable for the patients with BMI < 24. Further study is needed.

## Data Availability

The datasets used and/or analyzed during the current study are available from the corresponding author on reasonable request.

## Abbreviations

4D-CT: four-dimensional computed tomography
AP: Anterior-posterior
BMI: Body mass index
CBCT: Cone Beam Computed Tomography
CC: Cranial-caudal
GTV: Gross tumor volume
ITV: Internal tumor volume
KPS: Karnofsky performance status
ML: Medial-lateral
OAR: Organs at risk
PTV: Plan target volume
SBRT: Stereotactic radiotherapy
TMP: Thermoplastic masks
VCS: Vacuum cushion
